# Identification of NDUFAF1 in mediating K-Ras induced mitochondrial dysfunction by a proteomic screening approach

**DOI:** 10.18632/oncotarget.2968

**Published:** 2015-02-27

**Authors:** Peng Wang, Ming Song, Zhao-lei Zeng, Chao-feng Zhu, Wen-hua Lu, Jing Yang, Ming-zhe Ma, A-min Huang, Yumin Hu, Peng Huang

**Affiliations:** ^1^ Sun Yat-sen University Cancer Center, State Key Laboratory of Oncology in South China, Collaborative Innovation Center of Cancer Medicine, Guangzhou, China; ^2^ Department of Emergency Medicine, Sun Yat-sen Memorial Hospital, Guangzhou, China; ^3^ Department of Translational Molecular Pathology, The University of Texas M.D. Anderson Cancer Center, Houston, TX, USA

**Keywords:** K-Ras, mitochondria, NDUFAF1, glycolysis

## Abstract

Increase in aerobic glycolysis and mitochondrial dysfunction are important biochemical features observed in human cancers. Recent studies suggest oncogenic K-Ras can cause suppression of mitochondrial respiration and up-regulation of glycolytic activity through a yet unknown mechanism. Here we employed proteomic approach and used a K-Ras^G12V^ inducible cell system to investigate the impact of oncogenic K-Ras on mitochondria and cell metabolism. Mitochondria isolated from cells before and after K-Ras induction were subjected to protein analysis using stable isotope labeling with amino acids (SILAC) and liquid chromatography coupled with mass spectrometry (LC-MS). 70 mitochondrial proteins with significant expression alteration after K-Ras induction were identified. A majority of these proteins were involved in energy metabolism. Five proteins with significant decrease belong to mitochondrial respiratory chain complex I. NADH dehydrogenase 1 alpha subcomplex assembly factor 1 (NDUFAF1) showed most significant decrease by 50%. Such decrease was validated in primary human pancreatic cancer tissues. Knockdown of NDUFAF1 by siRNA caused mitochondrial respiration deficiency, accumulation of NADH and subsequent increase of glycolytic activity. Our study revealed that oncogenic K-Ras is able to induce significant alterations in mitochondrial protein expression, and identified NDUFAF1 as an important molecule whose low expression contributes to mitochondrial dysfunction induced by K-Ras.

## INTRODUCTION

Many cancer cells preferentially use glycolysis for generation of ATP and metabolic intermediates even in the presence of oxygen, and such metabolic feature is regarded as a hallmark of cancer [1]. Normal cells show active mitochondrial respiratory function and use the energy-efficient oxidative phosphorylation as the main route to generate ATP. The phenomenon of active aerobic glycolysis in cancer cells is known as Warburg effect, and is manifested as active glucose uptake and increased lactate production [2–4]. Such alterations in energy metabolism in cancer cells are often associated with mitochondrial dysfunction [5]. Importantly, recent studies suggest that metabolic alterations in cancer cells seem to be associated with activation of certain oncogenic signals (such as activating mutations in Ras oncogene) or loss of tumor suppressor function (such as loss or mutation of p53). Of note, the oncogenic Ras proteins including K-Ras, H-Ras and N-Ras are frequently mutated in human cancers. K-Ras is normally associated with the membrane lipid rafts and acts as an effector for signal transduction. Recent studies showed that K-Ras is able to translocate to mitochondria when phosphorylated [6]. Other studies showed that K-Ras is able to cause mitochondria dysfunction and regulate glucose metabolism [7–9], yet the detailed molecular mechanisms of mitochondria dysfunction induced by K-Ras remains to be investigated.

Recent advance in proteomic technology has enabled detail analysis of protein molecules that contribute to metabolic alterations in cancer and other diseases. Application of mitochondrial proteomics has shed new light on diagnosis and treatment of mitochondria dysfunction associated diseases, and allowed identification and quantification of biomarkers for the early diagnosis and pathologies [10, 11]. Stable isotope labeling with amino acids (SILAC) has become a pivotal tool to study changes in protein abundance in many different biological processes such as detection of biomarkers in tissue samples, the regulation of cell signaling, and the characterization of protein interactions. The current study used SILAC technology as initial screening for candidate proteins involved in mitochondrial dysfunction induced by K-Ras activating mutation (K-Ras^G12V^).

The main goals of this study were to identify mitochondrial proteins that may be involved in the metabolic alterations induced by constitutive activation of K-Ras, to investigate underlying mechanism, and to evaluate the clinical relevance. We have previously established a doxycycline inducible cell system with K-Ras^G12V^ expression vector (T-Rex/293), which induced mitochondrial dysfunction upon expression of K-Ras^G12V^ protein [9]. Since abnormal K-Ras activation and the associated mitochondrial dysfunction and metabolic shift are frequently observed in human cancer, the use of the current experimental system enabled us to identify a key molecular player that seems essential to maintain the metabolic changes in K-Ras-transformed cells. In this study, we isolated mitochondria from T-Rex/293 cells before and after induction of K-Ras^G12V^, followed by identification of mitochondrial proteins with significant changes using SILAC coupled with LC-MS analysis. A number of subunits of mitochondrial respiratory chain complex I were identified with significantly decreased protein ratios. We found that NDUFAF1 showed the most significant decrease after K-Ras^G12V^ induction and such decrease was also observed in clinical samples of pancreatic cancer tissues. The role of NDUFAF1 decrease in mediating mitochondrial dysfunction was further confirmed by siRNA knockdown, which led to inhibition of mitochondrial complex I activity and up-regulation of glycolysis.

## RESULTS

### K-Ras activation causes mitochondrial dysfunction

**Figure 1:**
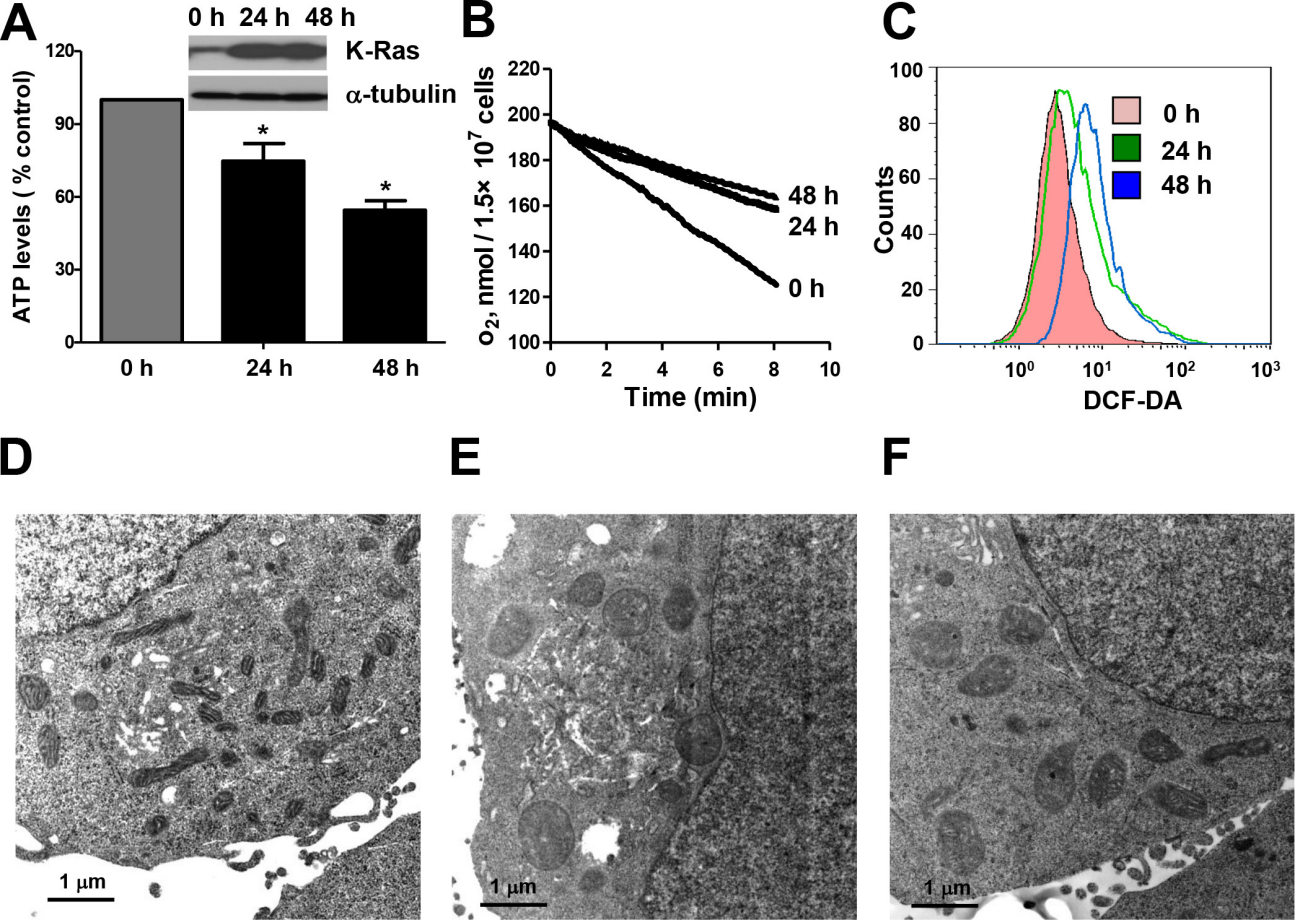
Expression of oncogenic K-Ras^G12V^ caused mitochondria dysfunction and metabolic alterations **(A)** ATP levels of T-Rex/293 cells before and after K-Ras^G12V^ induction by doxycycoine for 24 and 48 hrs. Data are shown as mean ± SD from three independent experiments, *P < 0.05. **(B)** Decrease of oxygen consumption rate after K-Ras^G12V^ induction for 24 and 48 hrs. **(C)** Increase of ROS level after K-Ras induction for 24 and 48 hrs detected by flow cytometry using DCF-DA. **(D–F)** Transmission electron microscopic analysis of mitochondria morphology. **(D)** Normal mitochondrial morphology of TRex/293 cell without K-Ras activation. **(E)** Alteration of mitochondrial morphology after K-Ras induction for 48 hrs. **(F)** Alteration of mitochondrial morphology after treatment of 100 nM rotenone for 36 hrs.

We have previously established a tetracycline-inducible K-Ras^G12V^ expression cell system [9]. In this system, expression of K-Ras^G12V^ protein can be induced by addition of doxycycline and a large portion of K-Ras^G12V^ protein is localized to the mitochondria, leading to changes of mitochondrial function (9). Since mitochondria play a critical role in carrying out oxidative phosphorylation and normally produces the majority of cellular ATP, we first measured ATP generation and oxygen consumption before and after K-Ras^G12V^ induction. As shown in Figure 1A, K-Ras^G12V^ expression caused a decrease of ATP generation in a time-dependent manner. Mitochondrial respiratory chain activity was also inhibited as evidenced by substantial decrease in oxygen consumption rate after K-Ras activation for both 24 hrs and 48 hrs (Figure 1B). The Seahorse XF analyser further demonstrated that K-Ras activation caused significant decrease in both maximal respiration and basal respiration levels (Supplemental Figure 1). One important biochemical event associated with oxidative phosphorylation is the production of reactive oxygen species (ROS) due to electron leakage from the respiratory chain. We found that K-Ras activation resulted in an increase of ROS production detected by DCF-DA (Figure 1C). Substantial elevation of intracellular superoxide (O_2_^−^) was also detected by MitoSOX Red mitochondrial superoxide indicator (Supplemental Figure 2A), indicating the source of ROS generation form mitochondrial electron leakage. We also examined the ultrastructure of mitochondria by transmission electron microscope (TEM). Normal tubular morphology of mitochondria was observed in control cells without K-Ras induction (Figure 1D). In contrast, swollen mitochondria were seen after K-Ras induction for 24 hrs (Figure 1E). Rotenone, a known inhibitor of mitochondrial respiratory chain complex I was used to treat the cells without K-Ras induction. As shown in Figure 1F, incubation with 100 nM rotenone for 36 h also caused alterations of mitochondrial morphology similar to that of cells with K-Ras^G12V^ induction. Taken together, these results suggest that K-Ras activation is able to cause significant alterations in mitochondrial function and ultrastructure.

### Proteomic analysis of mitochondrial proteins using SILAC

**Figure 2:**
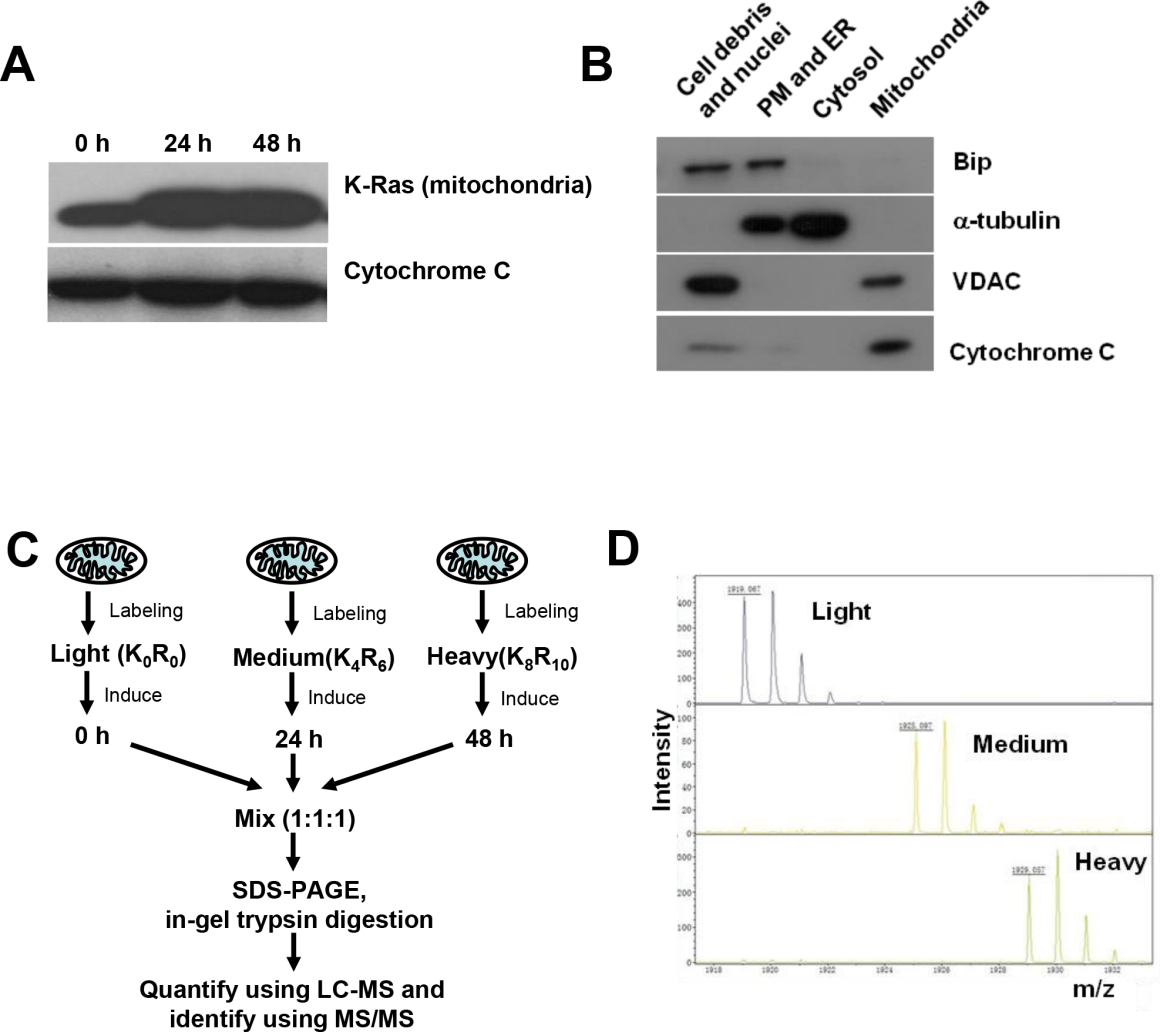
SILAC analysis of mitochondrial proteome in cells with and without K-Ras^G12V^ expression **(A)** Expression of K-Ras^G12V^ in mitochondria after doxycycline induction for 24 and 48 hrs. Cytochrome C was used as loading control for mitochondria extracts. **(B)** Purity of mitochondrial protein extracts by immunoblotting analysis. Cellular fractions were prepared from T-Rex/293 cells after K-Ras activation for 24 hrs. Purity of subcellular fractions was determined by immunoblotting analysis using antibodies against mitochondrial localized marker VDAC and cytochrome C, cytoplasmic localized marker α-tubulin, ER (endoplasmic reticulum) localized marker Bip. **(C)** Quantification and identification of mitochondrial proteins of T-Rex/293 cells before and after K-Ras induction by LC-MS. Cells were labeled with different isotopes as described in “Experimental procedures” under “Cell culture and isotopes labeling”. Light, control cells without K-Ras induction. Medium and Heavy, cells with K-Ras induction for 24 hrs and 48 hrs, respectively. The mixed mitochondrial lysates were separated by SDS-PAGE and analyzed by Mass Spectrometry. **(D)** The mass spectrum peaks indicates the separation of mitochondrial proteins labeled with different isotopes.

To identify the potential mitochondrial proteins involved in mediating mitochondrial dysfunction induced by K-Ras, we used quantitative proteomics to analyze mitochondria proteome dynamics using stable isotope labeling with amino acids in cell culture (SILAC). In consistence with our previous finding that K-Ras protein was associated with mitochondria, western blotting analysis confirmed the increase of K-Ras^G12V^ expression in a time-dependent manner in the mitochondrial fraction after addition of doxycyline (Figure 2A). The purity of mitochondria was validated by organelle specific protein markers of different subcellular fractions. As shown in Figure 2B, after induction for 24 hrs, the mitochondrial fraction was highly enriched in mitochondrial markers such as VDAC (Voltage-dependent anion channel) and cytochrome C, while other markers such as tubulin (cytosol marker) and Bip (endoplasmic reticulum marker) were absent in the mitochondrial fraction, indicating that there was no detectable contamination of other organelles.

Figure 2C depicts the proteomic strategy using SILAC and LC-MS for discovery of changes in mitochondrial proteins associated with metabolic alteration induced by K-Ras. The T-Rex/K-Ras cells with and without K-Ras induction were labeled with the indicated stable isotopes and cultured for 10 passages before protein isolation and LC-MS analyses. Control cells without K-Ras induction were cultured in isotope free medium (light density, K_0_R_0_). Cells with K-Ras induction for 24 hrs were cultured in [^13^C_6_]arginine and 4,4,5,5-D4-lysine labeled medium (Medium density, K_4_R_6_). Cells with K-Ras induction for 48 hrs were cultured in [^13^C_6_,^15^N_4_]arginine and [^13^C_6_,^15^N_2_]lysine labeled medium (Heavy density, K_8_R_10_). Mitochondria isolated from cells labeled with light, medium and heavy isotopes were mixed in a 1:1:1 ratio based on total protein concentrations. The mixed mitochondria were then lysed, properly digested to peptides, and then subjected to LC-MS/MS analysis. As shown in Figure 2D, the mass spectrum peaks of heavy and medium labeled proteins exhibited a corresponding shift according to their m/z values compared with light proteins, indicating proper incorporation of isotopes into the cells with different status of K-Ras as anticipated.

### Identification of K-Ras-induced alterations in mitochondrial proteins

**Figure 3:**
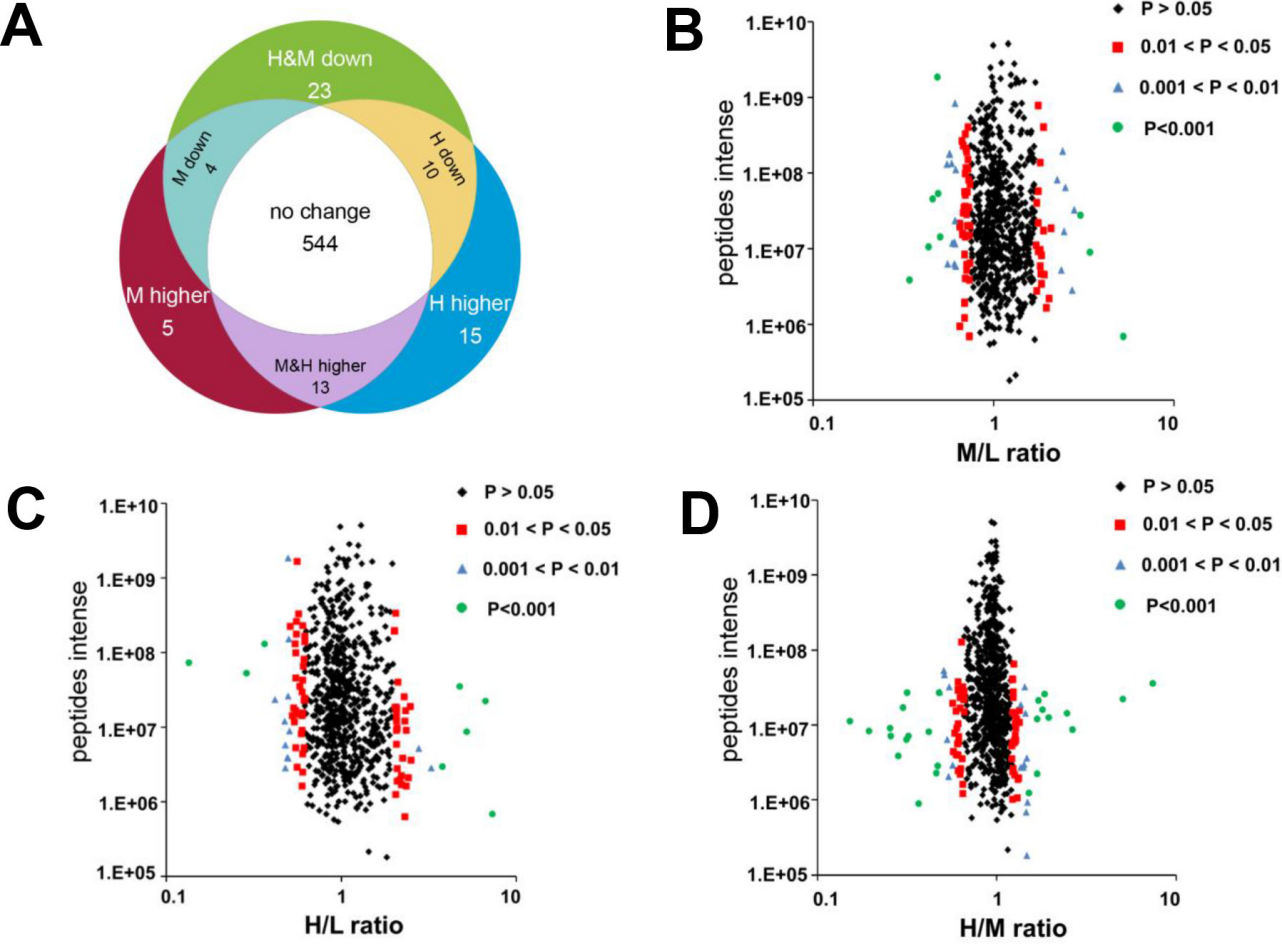
Effect of K-Ras^G12V^ activation on mitochondrial proteins expression profiles **(A)** Venn diagrams depict 614 proteins identified and quantified by SILAC and LC-MS. 544 proteins remained unchanged. 70 proteins showed significantly altered expression in cells with K-Ras induction for 24 or 48 hrs compared with control without K-Ras induction (*P* < 0.05). **(B–D)** Volcano plots showing protein ratios of each pair of cells indicated. H, K-Ras induction for 48 hrs. M, K-Ras induction for 24 hrs. L, control without K-Ras induction.

The mass spectrum analysis was used to characterize and quantify the mitochondrial proteomic changes induced by K-Ras^G12V^. As shown in Figure 3A, 614 mitochondria proteins were identified and quantified by LC-MS and SILAC. 544 mitochondrial proteins remained unchanged after K-Ras induction. 33 proteins showed a significantly increase and 37 proteins showed a significant decrease when compared with the control sample without K-Ras induction. Among the 33 proteins with increased expression, 5 proteins were upregulated only at the early time point (24 h); 15 proteins increased only at 48 h; and 13 proteins were upregulated at both 24 h and 48 h. Among the 37 proteins with lower expression, 4 proteins were downregulated at 24 h; 10 proteins were downregulated only after 48 hrs, and 23 proteins decreased at both 24 h and 48 h.

The protein density ratios (Figure 3B–3D) represent the relative abundance of proteins in cells with K-Ras induction compared with cells without induction (H/L for 48 h, M/L for 24 h), or cells with 48 hr induction compared with cells with 24 hr induction (H/M). The majority of proteins remained unchanged with the density distribution at 1:1 ratio. A small number of proteins showed significant change (*P* < 0.05), and suggested a possibility that these mitochondrial proteins might contribute to the mitochondrial dysfunction and metabolic alterations induced by K-Ras activation. As shown in Table 1, K-Ras was identified in the mitochondrial proteome and its expression increased by 5.2 and 7.3 folds after induction for 24 and 48 hs, respectively (“M&H higher”, line 12). This was consistent with the findings in Figure 2A showing the increase of K-Ras protein in mitochondria, and thus served as a good validation of this proteomic analysis. We then used Panther (protein analysis through evolutionary relationships) analysis to investigate the biological pathways involved after K-Ras activation. As shown in Table 2, we identified 13 groups of proteins based on the biological function of these differentially expressed proteins after K-Ras induction. Of particular note, among the approximately 70 mitochondrial proteins that showed altered expression after K-Ras induction, 9 proteins are involved in generation of metabolite and energy precursors and 36 proteins are associated with metabolic process (Table 2 lines 9 and 10, respectively).

**Table 1:** Differential expression of mitochondrial proteins after K-ras activation

Protein IDs	Protein Names	Gene Names	Ratio M/L Normalized	Ratio M/L *p*-value	Ratio H/L Normalized	Ratio H/L *p*-value
M&H higher						
IPI00024551	UPF0480 protein C15orf24	C15orf24	2.405	0.005	1.998	0.049
IPI00784366	AP-2 complex subunit beta-1	AP2B1	1.954	0.026	2.338	0.021
IPI00059762	Lysophospholipase-like protein 1	LYPLAL1	3.416	0.001	2.352	0.021
IPI00908804	highly similar to ATP-binding cassette sub-family D member 3	ABCD3	2.444	0.005	2.319	0.022
IPI00294472	Transmembrane emp24 domain-containing protein 5	TMED5	2.075	0.017	2.029	0.045
IPI00328840	THO complex subunit 4	THOC4	1.882	0.034	2.069	0.041
IPI00305289	Kinesin-like protein KIF11	KIF11	2.104	0.015	2.409	0.018
IPI00215637	ATP-dependent RNA helicase DDX3X	DDX3X	1.806	0.044	2.082	0.040
IPI00018246	Hexokinase-1	HK1	2.727	0.002	3.268	0.002
IPI00879004	DNA topoisomerase 2-alpha	TOP2A	2.374	0.006	2.753	0.008
IPI00034159	V-type proton ATPase subunit d 1	ATP6V0D1	1.803	0.045	2.069	0.041
IPI00423570	GTPase Kras	KRAS	5.210	0.001	7.269	0.001
IPI00293073	Mitofusin-1	MFN1	0.696	0.035	4.734	0.001
H higher						
IPI00031691	60S ribosomal protein L9	RPL9	1.737	0.056	2.107	0.037
IPI00419258	High mobility group protein B1protein B1	HMGB1	1.755	0.053	2.061	0.042
IPI00026111	Transmembrane and coiled-coil domain-containing protein 1	TMCO1	1.599	0.090	2.060	0.042
IPI00376377	Dehydrogenase/reductase (SDR family) member 2	DHRS2	1.493	0.128	2.076	0.040
IPI00374657	Putative uncharacterized protein VAPA	VAPA	1.637	0.079	2.162	0.033
IPI00418169	Putative uncharacterized protein DKFZp686P03159	ANXA2	1.395	0.175	2.468	0.016
IPI00293564	Hydroxymethylglutaryl-CoA lyase, mitochondrial	HMGCL	1.032	0.485	3.781	0.001
IPI00554481	4F2 cell-surface antigen heavy chain	SLC3A2	1.611	0.086	2.490	0.015
IPI00329600	Probable saccharopine dehydrogenase	SCCPDH	1.271	0.256	2.036	0.044
IPI00293845	Telomere-associated protein RIF1	RIF1	1.490	0.129	2.301	0.023
IPI00478810	Ribosomal protein S10	RPS10	0.928	0.328	2.279	0.025
IPI00293260	DnaJ homolog subfamily C member 10	DNAJC10	1.453	0.145	2.059	0.042
IPI00003856	V-type proton ATPase subunit E 1	ATP6V1E1	1.557	0.103	5.208	0.001
IPI00306667	2′,3′-cyclic-nucleotide 3′-phosphodiesterase	CNP	1.335	0.210	6.639	0.001
M higher						
IPI00418414	Hexaprenyldihydroxybenzoate methyltransferase, mitochondrial	COQ3	10.682	0.001	1.407	0.204
IPI00644079	Heterogeneous nuclear ribonucleoprotein U	HNRNPU	1.833	0.040	1.660	0.111
IPI00604590	Nucleoside diphosphate kinase	NME1-NME2	3.010	0.001	1.376	0.219
IPI00005202	Membrane-associated progesterone receptor component 2	PGRMC2	2.794	0.001	1.936	0.057
IPI00291939	Structural maintenance of chromosomes protein 1A	SMC1A	2.020	0.021	1.527	0.153
H&M down						
IPI00456965	Ubiquinone biosynthesis methyltransferase COQ5, mitochondrial	COQ5	0.641	0.014	0.479	0.006
IPI00015808	Nucleolar GTP-binding protein 2	GNL2	0.490	0.001	0.286	0.001
IPI00293975	Glutathione peroxidase 1	GPX1	0.678	0.026	0.596	0.038
IPI00893857	NADH dehydrogenase 1 alpha subcomplex subunit 11	NDUFA11	0.615	0.008	0.478	0.006
IPI00020050	Probable ATP-dependent RNA helicase DDX28	DDX28	0.716	0.047	0.498	0.008
IPI00026512	GTP-binding protein era homolog	ERAL1	0.644	0.015	0.418	0.001
IPI00847172	Peripheral-type benzodiazepine receptor	PBR	0.589	0.005	0.473	0.005
IPI00032872	28S ribosomal protein S16, mitochondrial	MRPS16	0.695	0.035	0.568	0.027
IPI00005966	NADH dehydrogenase 1 alpha subcomplex subunit 12	NDUFA12	0.662	0.020	0.554	0.021
IPI00219772	NADH dehydrogenase 1 beta subcomplex subunit 7	NDUFB7	0.679	0.027	0.578	0.030
IPI00470631	Ubiquinone biosynthesis protein COQ9, mitochondrial	COQ9	0.708	0.042	0.546	0.019
IPI00290614	Endonuclease G, mitochondrial	ENDOG	0.609	0.007	0.537	0.016
IPI00514501	Chromosome 1 open reading frame 57	C1orf57	0.693	0.034	0.546	0.019
IPI00333763	Glutaredoxin-related protein 5	GLRX5	0.584	0.004	0.545	0.019
IPI00024742	Cytochrome b-c1 complex subunit 8	UQCRQ	0.674	0.025	0.599	0.040
IPI00176469	Chaperone activity of bc1 complex-like, mitochondrial	CABC1	0.502	0.000	0.593	0.037
IPI00010244	28S ribosomal protein S11, mitochondrial	MRPS11	0.571	0.003	0.553	0.021
IPI00023673	Galectin-3-binding protein	LGALS3BP	0.486	0.001	0.494	0.008
IPI00890773	Protein MTO1 homolog, mitochondrial	MTO1	0.642	0.014	0.559	0.023
IPI00738524	General transcription factor IIH subunit 2-like protein	GTF2H2	0.014	0.001	0.134	0.001
IPI00021785	Cytochrome c oxidase subunit 5B, mitochondrial	COX5B	0.610	0.007	0.615	0.048
IPI00152685	Tetratricopeptide repeat protein 15	TTC15	0.643	0.014	0.497	0.008
IPI00219381	NADH dehydrogenase 1 alpha subcomplex subunit 2	NDUFA2	0.458	0.001	0.549	0.020
H down						
IPI00448630	Sterile alpha and TIR motif-containing protein 1	SARM1	1.096	0.415	0.591	0.036
IPI00032560	Complex I intermediate-associated protein 30	NDUFAF1	0.876	0.235	0.503	0.009
IPI00013679	Deoxyuridine 5′-triphosphate nucleotidohydrolase	DUT	0.868	0.223	0.491	0.007
IPI00217081	FUN14 domain-containing protein 1	FUNDC1	0.855	0.202	0.559	0.023
IPI00419626	39S ribosomal protein L55, mitochondrial	MRPL55	1.201	0.312	0.599	0.040
IPI00167638	GTP-binding protein 10	GTPBP10	0.782	0.105	0.600	0.040
IPI00217871	Delta-1-pyrroline-5-carboxylate dehydrogenase	ALDH4A1	0.993	0.451	0.594	0.038
IPI00008483	Amine oxidase A	MAOA	0.800	0.126	0.547	0.019
IPI00037448	Glyoxylate reductase/hydroxypyruvate reductase	GRHPR	0.902	0.280	0.596	0.038
IPI00011276	2-oxoisovalerate dehydrogenase subunit beta	BCKDHB	0.729	0.056	0.560	0.023
M down						
IPI00016443	Protein EMI5 homolog, mitochondrial	C11orf79	0.433	0.001	0.830	0.269
IPI00830136	Uncharacterized protein C1orf31	C1orf31	0.692	0.033	1.025	0.468
IPI00215790	60S ribosomal protein L38	RPL38	0.647	0.015	1.191	0.332
IPI00879060	2-amino-3-ketobutyrate coenzyme A ligase, mitochondrial	GCAT	0.650	0.016	1.198	0.327

H (K-ras induction for 48 hrs), M (K-ras induction for 24 hrs), L (control without induction).

**Table 2:** Percentage of identified mitochondrial proteins based on their biological functions

	Category name (Accession)	Counts	Percent of total genes
1	Cell communication (GO:0007154)	8	11.60%
2	Cellular process (GO:0009987)	14	20.30%
3	Transport (GO:0006810)	13	18.80%
4	Cellular component organization (GO:0016043)	1	1.40%
5	Apoptosis (GO:0006915)	3	4.30%
6	System process (GO:0003008)	1	1.40%
7	Response to stimulus (GO:0050896)	2	2.90%
8	Developmental process (GO:0032502)	2	2.90%
9	Generation of precursor metabolites and energy (GO:0006091)	9	13.00%
10	Metabolic process (GO:0008152)	36	52.20%
11	Cell cycle (GO:0007049)	4	5.80%
12	Immune system process (GO:0002376)	3	4.30%
13	Cell adhesion (GO:0007155)	4	5.80%

Because K-Ras activation caused a significant decrease in cellular oxygen consumption and ATP production (Figure 1), we further analyzed the group of 9 proteins involved in the generation of precursor metabolites and energy in order to identify the mitochondrial proteins contributing to K-Ras-induced metabolic alterations. Out of the 9 proteins in this group, 5 proteins belonged to the mitochondrial respiratory chain complex I, one protein was a subunit of complex III, and one belonged to complex IV (Table 3).

**Table 3:** Differential expression of mitochondrial respiratory chain subunits after K-ras induction for 48 hrs

Protein IDs	Gene Names	Protein description	Mitochondria location	Ratio H/L Normalized	Ratio H/L *p* value
IPI00032560	NDUFAF1	NADH dehydrogenase 1 alpha subcomplex, assembly factor 1	Complex I	0.50267	0.01
IPI00893857	NDUFA11	NADH dehydrogenase 1 alpha subcomplex subunit11	Complex I	0.47817	0.01
IPI00219381	NDUFA2	NADH dehydrogenase 1 alpha subcomplex subunit 2	Complex I	0.54878	0.02
IPI00005966	NDUFA12	NADH dehydrogenase 1 alpha subcomplex subunit 12	Complex I	0.55382	0.02
IPI00219772	NDUFB7	NADH dehydrogenase 1 beta subcomplex subunit 7	Complex I	0.578	0.03
IPI00024742	UQCRQ	Complex III subunit 8	Complex III	0.59858	0.04
IPI00021785	COX5B	Cytochrome c oxidase subunit 5B, mitochondria	Comolex IV	0.6149	0.04

### Down regulation of NDUFAF1 in K-Ras-transformed cells and in pancreatic cancer tissues

**Figure 4:**
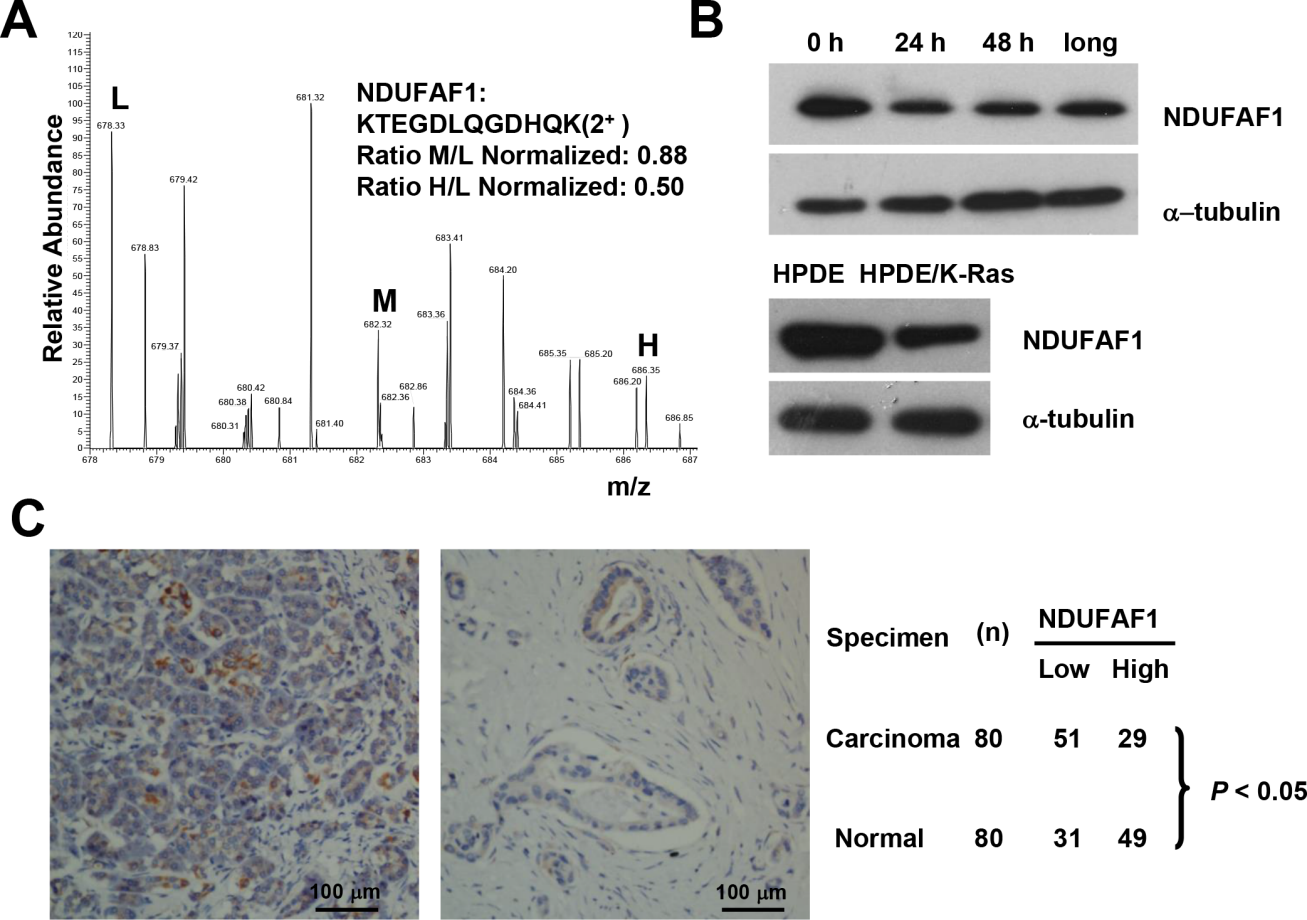
Suppression of NDUFAF1 expression by K-Ras^G12V^ and its low expression in pancreatic cancer tissues **(A)** Quantification of NDUFAF1 proteins by mass spectrum from three samples. L, control without K-Ras induction. M, K-Ras induction for 24 hrs. H, K-Ras induction for 48 hrs. **(B)** Westernblot detection of NDUFAF1 protein in T-Rex/293 cells before and after K-Ras induction and comparison of NDUFAF1 protein in the K-Ras transformed human pancreatic ductal epithelial (HPDE) cells and the parental control. Long, K-Ras induction >1 month. **(C)** Representative immunohistochemical staining of tissue microarray showing NDUFAF1 expression is significantly down regulated in pancreatic cancer tissues. Left panel, normal pancreatic duct; Right panel, pancreatic ductal carcinoma. The comparison of NDUFAF1 expression between normal and cancer tissue was analyzed with Fisher's exact test, *P* < 0.05. *n* = 80.

The results shown in Table 3 suggest that down-regulation of certain complex I components, especially NADH dehydrogenase (ubiquinone) 1 alpha sub-complex assembly factor 1 (NDUFAF1), might play an important role in mediating mitochondrial dysfunction induced by K-Ras activation, since the expression of these protein subunits were significantly down-regulated. It is known that NDUFAF1 plays a key role in the assembly of mitochondrial complex I [12–14]. LC-MS analysis showed that the expression of NDUFAF1 was down-regulated by approximately 20% and 50% after K-Ras induction for 24 hrs and 48 hrs, respectively (Figure 4A). Western blot analysis also demonstrated a significant decrease of NDUFAF1 expression after K-Ras induction at both time points (24 and 48 h), and such decrease was consistently observed in cells with long-term (>1 month) K-Ras induction (Figure 4B). Importantly, doxycycline induced K-Ras*^G12V^* expression in a dose-dependent manner, and the level of NDUFAF1 protein decreased as the expression of K-Ras increased. In addition, K-Ras expression caused a decrease in NDUFAF1 protein under hypoxia condition, which mimics the tumor microenvironment (Supplemental Figure 1). The expression of NDUFAF1 was also tested in K-Ras transformed human pancreatic ductal epithelial (HPDE/K-Ras) cells established previously [15], and the immunoblotting results showed that NDUFAF1 was significantly decrease in the transformed cells compared with the non-transformed parental HPDE cells (Figure 4B, lower panel).

Considering that oncogenic mutations of K-Ras are frequently observed in the vast majority of human pancreatic cancer cases [16], we next tested if the findings from our proteomic analysis in cell lines could be verified in pancreatic tumor samples, using a pancreatic cancer tissue arrays containing 80 pairs of pancreatic ductal carcinoma and the adjacent normal pancreatic tissues. As shown in Figure 4C, immunohistochemical analysis revealed that the majority 64% (51/80) of the pancreatic cancer tissues exhibited low expression of NDUFAF1. In contrast, the majority of the adjacent normal pancreatic tissues 61% (49/80) showed high expression of NDUFAF1. Overall, there was a statistically lower expression of NDUFAF1 in pancreatic carcinoma than in normal pancreatic tissues (*P* < 0.05, Fisher's exact test), suggesting that a decrease in NDUFAF1 expression may be clinically relevant in pancreatic cancer.

### Role of NDUFAF1 down-regulation in mediating mitochondrial dysfunction induced by K-Ras

**Figure 5:**
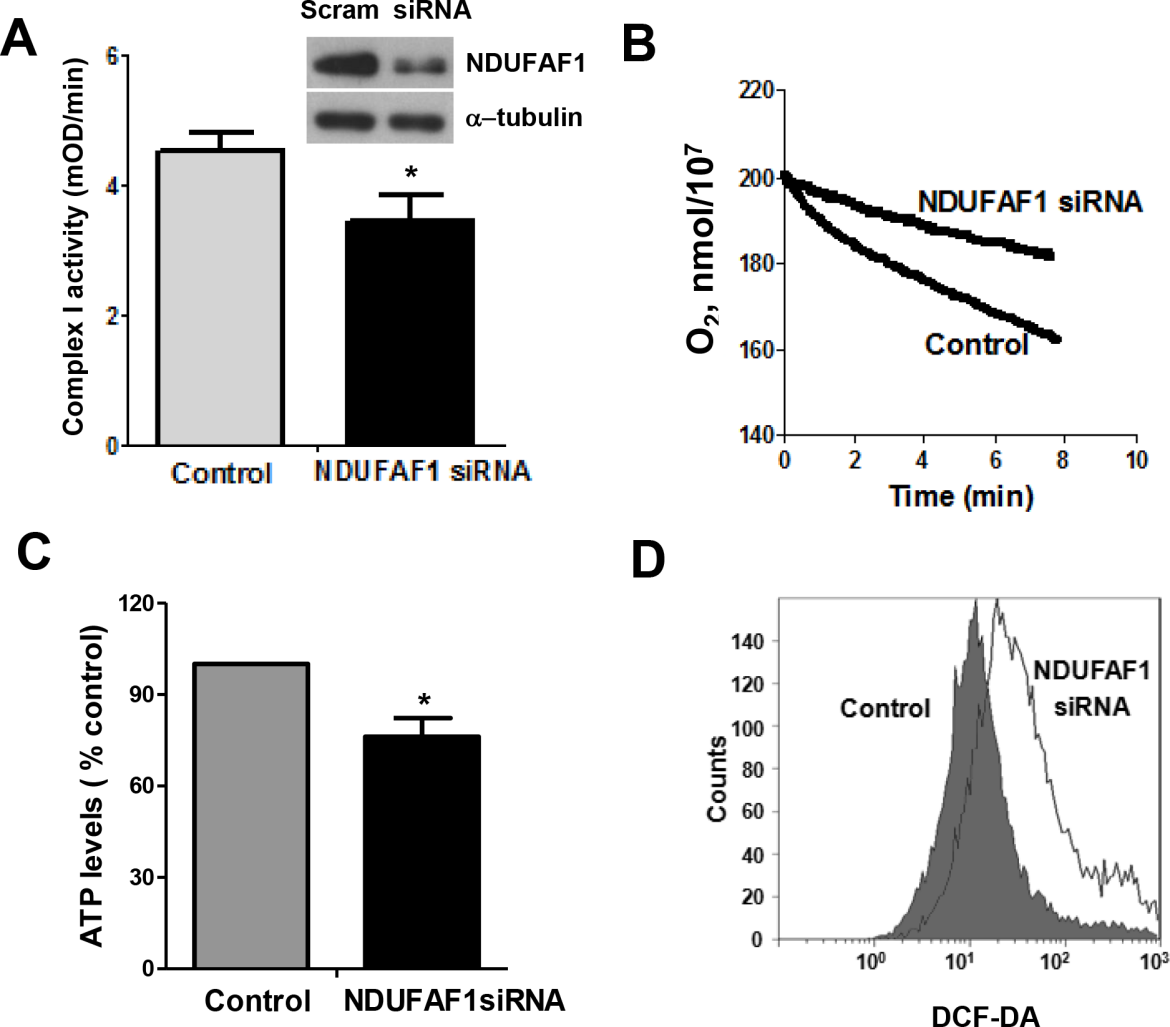
Knockdown of NDUFAF1 causes mitochondrial dysfunction **(A)** Inhibition of mitochondrial respiratory chain complex I activity by knockdown of NDUFAF1. Data are shown as mean ± SD, *P < 0.05. Western blot analysis showing knockdown efficiency of NDUFAF1. Non-targeting control siRNA (Scram) was used as control. **(B)** Decrease of oxygen consumption rate after knockdown of NDUFAF1. **(C)** Decrease of ATP production after NDUFAF1 knockdown, *P < 0.05. **(D)** Detection of ROS level before and after knockdown of NDUFAF1 by flow cytometry using CM-DCFDA.

To further test the functional consequence of NDUFAF1 down-regulation, we used siRNA to specifically knock down the expression of NDUFAF1 in T-Rex/293 cells. As shown in Figure 5A, siRNA against NDUFAF1 effectively suppressed its expression, leading to a significant decrease of the activity of mitochondrial respiratory chain complex I. Because NDUFAF1 is a complex I assembly factor, a Blue-native Polyacrylamide Gel Electrophoresis (BN-PAGE) was performed to detect the effect of knockdown of NDUFAF1 or K-Ras activation. Both K-Ras activation and knockdown of NDUFAF1 mainly inhibited the assembly of complex I (Supplemental Figure 3). As a result, the mitochondrial respiratory chain activity was significantly inhibited, as evidenced by a decrease in oxygen consumption rate (Figure 5B). Because mitochondrial respiratory chain is the major site of ATP generation and reactive oxygen species (ROS) production, we also analyzed the possible metabolic alterations after NDUFAF1 knockdown. Suppression of NDUFAF1 expression by siRNA caused a significant decrease in ATP generation (Figure 5C) and a substantial increase of ROS level detected by both DCF-DA (Figure 5D) and MitoSOX (Supplemental Figure 2B). Taken together, these data suggest that suppression of NDUFAF1 expression could cause significant mitochondrial dysfunction.

**Figure 6:**
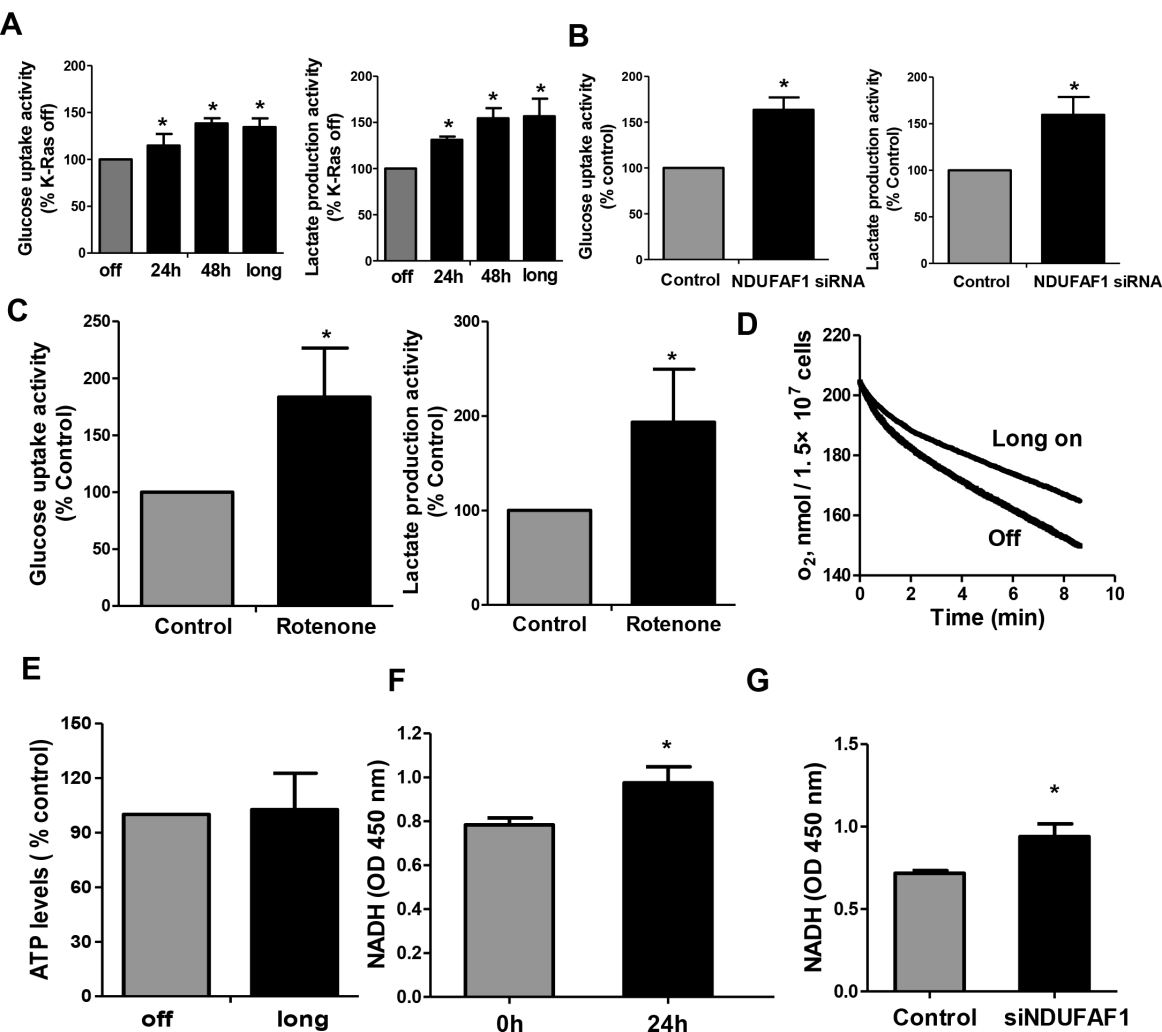
Mitochondrial dysfunction leads to increase of glycolysis activity and accumulation of NADH in T-Rex/293 cells **(A–B)** K-Ras activation and knockdown of NDUFAF1 caused increase of glycolytic activity as measured by glucose uptake and lactate production. Long, K-Ras induction for more than 1 month by addition of doxycycline. **(C)** Inhibitor of mitochondrial respiratory chain complex I by rotenone (10 nM) caused increase of glycolytic activity in TRex/293 cells. **(D)** Oxygen consumption remained inhibited after long term induction of K-Ras (>1 month). **(E)** ATP production remained unchanged after long term induction of K-Ras. **(F–G)** Activation of K-Ras for 24 h and knockdown of NDUFAF1 caused increase of NADH generation.

We then further analyzed the effect of K-Ras-mediated mitochondrial dysfunction on glycolytic activity. Figure 6A shows a significant elevation of glycolytic activity in T-Rex/293 cells after short-term (24–48 h) or long term (>1 month) induction of K-Ras expression. Since our mitochondrial proteomic analysis identified NDUFAF1 as an important protein suppressed by K-Ras, we tested if knockdown of NDUFAF1 could also cause increase of glycolytic activity. In agreement with the metabolic alterations induced by K-Ras, suppression of NDUFAF1 expression also caused a significant increase in glycolysis, as revealed by the increase in glucose uptake and lactate production (Figure 6B). To further confirm that the increased of glycolysis is a consequence of mitochondrial dysfunction, we used rotenone, a specific inhibitor of mitochondria complex I, to test its effect on glycolysis. As shown in Figure 6C, incubation of T-Rex/293 cells with 10 nM rotenone for 12 hrs caused a significant increase in glucose uptake and lactate production, comparable to that seen in the T-Rex/293 cells with NDUFAF1 knockdown by siRNA (Figure 6B).

The above results showed that acute induction of K-Ras caused mitochondrial dysfunction and metabolic switch to glycolysis. We then further analyzed the metabolic state of cells after long-term induction of K-Ras. Mitochondrial respiratory chain activity remained inhibited in T-Rex/293 cells with continuous induction of K-Ras for more than 1 month (Figure 6D), while glycolytic activity also remained upregulated (Figure 6A, column indicated with “long”). Interestingly, in contrast to the acute ATP decrease after K-Ras induction for 24–48 hrs, the cellular ATP level recovered to normal levels after long-term induction of K-Ras for more than 1 month (Figure 6E), suggesting the eventual metabolic adaptation that compensate ATP generation.

NADH is the main substrate for mitochondrial respiratory chain, and mitochondrial dysfunction could cause an accumulation of NADH. NADH is required for regeneration of NAD^+^, which is important to maintain active glycolysis [17]. Indeed, we observed that activation of K-Ras for 24 hrs (Figure 6F) or suppression of NDUFAF1 by siRNA (Figure 6G) led to an increase of NADH by approximately 20% in T-Rex/293 cells. Together, these data suggest that down-regulation of NDUFAF1 might be an important event contributing to K-Ras-induced mitochondrial dysfunction and increase in glycolysis.

## DISCUSSION

K-Ras represents the most frequent mutation during malignant transformation and cancer development [18]. Increasing evidence suggests a strong association between oncogenic transformation and metabolic alterations including mitochondrial dysfunction and upregulation of aerobic glycolysis [8, 19, 20]. Our previous study demonstrated that activation of mutated K-Ras (G12V) inhibited the function of mitochondrial respiratory chain, leading to an increase of glycolytic activity [9]. In order to thoroughly understand the molecular mechanisms associated with K-Ras oncogenic signaling and alterations in cellular metabolism, the current study used a subcellular proteomic strategy to identify changes of proteins in the mitochondria of K-Ras transformed cells with SILAC/LC-MS analysis.

By comparing the mitochondrial proteome of T-Rex/293 cells before and after K-Ras induction in the doxycyline inducible cell system, we were able to detect 614 mitochondrial proteins, and among them we identified 70 proteins with significant altered abundance. The biological function analysis revealed that 65% of the altered mitochondrial proteins were associated with generation of metabolites and metabolic process. Intriguingly, we found that 7 out of the 70 proteins with change in expression were subunits of the mitochondrial respiratory chain complexes, and 5 out of 7 subunits identified belong to complex I.

Mitochondria are the major source of cellular ATP production. The mitochondrial respiratory chain produces ATP through oxidative phosphorylation. Complex I (NADH dehydrogenase) initiates the electron transportation by oxidizing NADH to NAD^+^. Our results showed that a majority of the complex I subunits including NDUFA2, NDUFA11, NDUFA12, NDUFB7, NDUFAF1 were substantially down-regulated (50%) after K-Ras^G12V^ induction (Table 3), consistent with our previous finding that K-Ras activation inhibited activity of complex I [9]. K-Ras mutation is frequently observed in pancreatic cancer. Our immunohistochemistry analysis revealed that the majority of pancreatic carcinoma tumor tissues exhibited lower expression of NDUFAF1 compared with the normal tissue, suggesting that the down-regulation of NDUFAF1 is likely clinically relevant. Our results also suggest that disruption of complex I may play a major role in mediating suppression of mitochondrial respiration induced by K-Ras^G12V^. Although K-Ras represents the most frequent mutation observed in human cancer, several mutations to Ras can transform normal cells to cancer. While K-Ras mainly affects complex I assembly of mitochondrial respiratory chain, the effect of H-ras on mitochondrial respiration remain to be determined. Yang et al. showed that oncogenic H-ras^Q61L^ transformation caused defects of mitochondrial respiration in NIH-3T3 cells [21]. In contrast, Telang et al. demonstrated that introduction of activated H-ras^V12^ into immortalized human bronchial epithelial cells increased tricarboxylic acid cycle activity, oxygen consumption and energetic reliance on electron transport. These contrasting models suggest that the effect of oncogenic Ras on mitochondrial respiration may be context dependent.

However, the precise mechanism of inhibition of NDUFAF1 by K-Ras activation remains unclear at the present time. Ecsit is a cytosolic adaptor protein essential for inflammatory response. It has been reported that Ecsit can localize to mitochondria where it interacts with chaperone NDUFAF1 and functions in complex I assembly. The knockdown of Ecsit in mitochondria results in NDUFAF1 decrease and impaired complex I assembly [22]. Our previous studies have demonstrated translocation of K-Ras protein to mitochondria [9]. It would be interesting to further study the possible interaction of K-Ras and Ecsit and the effect on complex I assembly. In addition, the sequence analysis of human NDUFAF1 promoter revealed several putative TR4-hormone response elements and a chromatin immunoprecipitaiton (ChIP) assay has demonstrated NDUFAF1 is a directly target of TR4 [23]. The potential regulation of TR4 by K-Ras is worth further study.

The association of oncogenes and upregulation of glycolysis has been proposed for some time, although the underlying molecular mechanism still remains to be investigated. Because complex I initiates the electron transportation by oxidizing NADH to NAD^+^, here we found that inhibition of NDUFAF1 by activation of K-Ras resulted in accumulation of NADH. Because reoxidization of NADH is required to maintain proper NADH/NAD^+^ ratio and redox homeostasis, the accumulated NADH after K-Ras activation could be converted to NAD^+^ by redox-regulatory enzymes such as NAD(P)H Oxidase (NOX). Indeed, the regeneration of NAD^+^ is important to maintain active glycolysis. Our previous studies demonstrated that K-Ras activation or dysfunction of mitochondrial leads to up-regulation of NOX [9, 17]. Overexpression or elevated activity of NOX has also been reported in various cancer types including pancreatic cancer [17, 24, 25]. Although the focus of Warburg effect has been energy-centric, recent studies have suggested that the reprogrammed metabolism may be essential for macromolecular biosynthesis [26]. For instance, the tumor-specific form of pyruvate kinase (PKM2) increases the incorporation of glucose carbons into lipids [27]. Ying et al. reported that KrasG12D enhanced glycolytic flux into the pentose phosphate pathway without affecting TCA cycle [28]. Therefore, the decrease of NDUFAF1 observed in our model and pancreatic cancer specimen may indicate metabolic reprogramming driven by K-Ras oncogenic activation.

In summary, this study used SILAC analysis to reveal mitochondrial proteomic changes associated with mitochondrial dysfunction and metabolic alterations induced by oncogenic K-Ras^G12V^. Our results suggest that inhibition of mitochondrial respiratory chain complex I may significantly contribute to mitochondrial dysfunction and up-regulation of glycolytic activity during K-Ras-mediated transformation and metabolic “reprogramming”. Suppression of NDUFAF1 expression and down regulation of other mitochondrial respiratory chain complex components may be important events contributing to K-Ras-induced mitochondrial dysfunction. The mechanisms by which K-Ras activation leads to down-regulation of mitochondrial respiratory chain components currently remain unclear and require further investigation.

## METHODS

### Cell culture and stable isotope labeling

The doxycycline inducible T-Rex/293 cells were established as previously described [9]. T-Rex/K-Ras cells were cultured in Dulbecco's modified Eagle's medium supplemented with 10% tetracycline free fetal bovine serum. The medium without isotope and labeled with L-Arginine (R0) (84 mg/ml) or L-lysine (K0) (146 mg/ml) was considered as “light” (L) medium. The medium containing L-[^13^C_6_] arginine (R6) and L-4,4,5,5-D_4_-lysine (K4) (Sigma Aldrich) was considered as “medium” (M) medium. The medium containing L-[^13^C_6_,^15^N_4_] arginine (R10) and L-[^13^C_6_,^15^N_2_] lysine (K8) was considered as “heavy” (H) medium. T-Rex/K-Ras cells were cultured in SILAC medium for ten passages to ensure complete incorporation of isotopic amino acids. Cells were then induced by doxycycline for 24 hrs in M medium and 48 hrs in H medium. Cells without addition of doxycyline were cultured in L medium. HPDE cells and HPDE/K-Ras cells were cultured in DMEM/F12 medium.

### Mitochondria isolation

Mitochondria of T-Rex/293 cells were isolated using Q-proteome Mitochondria Isolation kit (Qiagen). Briefly, cells were washed and re-suspended in lysis buffer and centrifuged at 1000 × g for 10 mins. The pellet was then re-suspended in Disruption Buffer and passed through a syringe 10 times, and re-centrifuged at 1000 × g for 10 mins to remove the nuclei, cell debris, and unbroken cells. The supernatant containing mitochondria and the microsomal fraction was centrifuged at 6000 × g for 10 mins to collect mitochondria pellet. After removal of the supernatant, the crude mitochondria pellet was re-suspended in Mitochondria Purification Buffer. The purified mitochondria were isolated by density gradient centrifugation. Finally, the mitochondria were re-suspended in lysis buffer containing 8 M urea, 4% CHAPS, 65 mM DTT, and 40 mM Tris and sonicated at 100 watts for 30 s. The sample was then centrifuged at 25,000 × g for 30 mins and the supernatant was collected as the mitochondrial protein lysates.

### LC-MS analysis

Same amount of mitochondria labeled with different isotypes were mixed and separated by one-dimensional SDS-PAGE. The gel was stained with coomassie blue R-250 and cut into 40 slices. The gel slices were then subjected to in-gel digestion with trypsin. Extraction and concentration of the samples for MS analysis were carried out as described previously [29, 30].

Trypsin-digested peptides were separated by HPLC system (Thermo Scientific). The sample was eluted onto a C_18_ nanocolumn (0.1 mm inner diameter × 10 cm long, Michrom Bioresources) using 0.1% formic acid in water as mobile phase A and 0.1% formic acid in 80% acetonitrile as mobile phase B. A linear gradient of 5–35% solvent B (90% acetonitrile with 0.1% formic acid) was eluted over 120 mins with a constant flow of 300 nl/min. The HPLC system was coupled to a linear ion trap-orbitrap hybrid mass spectrometer (LTQ-OrbitrapXL, Thermo Scientific) via a nanoelectrospray ion source (Proxeon Biosystems). The spray voltage was 1.2 kV, and the temperature of the heated capillary was 200°C. The full-scan mass range was from m/z 400–2000 with resolution 60,000 at m/z 400. The five most intense ions were sequentially isolated for fragmentation in the linear ion trap by collision-induced dissociation. Maximal filling times were 1,000 ms for the full scans and 150 ms for the MS/MS scans. The dynamic exclusion list was restricted to a maximum of 500 entries with a maximum retention period of 90 s and a relative mass window of 10 ppm.

### MS data analysis

The MS/MS peak list was generated by Xcalibur software (Thermo Scientific) and analyzed with MaxQuant (Version, 1.0.13.13). The output files were submitted to Mascot (Version 2.2, Matrix Science) for peptide and protein identification. Searches were conducted against target-decoy human MaxQuant (ipi.HUMAN.v3.52.decoy). Enzyme specificity was set to that of trypsin, allowing for N-terminal cleavage to proline and between aspartic acid and proline. Searches were performed with a MS tolerance of 20 ppm and a fragment tolerance of 0.5 Da. Carboxyamidomethylated cysteine was used as a fixed modification. Variable modifications included methionine oxidation and protein N-terminal acetylation. Peptides with minimum of 6 amino acids were allowed for analysis and proteins were identified if they had at least one unique peptide. The results were reported with 5% peptide false discovery rate and 1% protein false discovery rate. The posterior error probabilities (PEP, false hit probability given the peptide score and length) should be below or equal to 0.01. The sequences corresponding to the 760 protein entries were searched in the database.

### Antibodies and RNA interference

The following antibodies were used for immunoblotting analysis: K-Ras, Cytochorme C and NDUFAF1 (Abcam), tubulin, VDAC and Bip (Cell Signaling). The siRNA (Ribobio, Guangzhou, China) target sequence for NDUFAF1 was 5′-GCAAGGAGATCACCAGAAA-3′. A non-specific scramble siRNA was used as control.

### Transmission electron microscope

Samples were prepared as described previously [31]. Briefly, cells were fixed in 2.5% glutaraldehyde for 2 hours and 1% osmium tetroxide for another 1 hour. Cells were then dehydrated in a graded series of ethanol and embedded in spurr resin. Sections were prepared and examined with Morgagni transmission electron microscope (FEI).

### Immunohistochemistry

Immunohistochemical staining for NDUFAF1 was performed on the pancreatic cancer tissue microarray (TMA, Shanghai Biochip, Shanghai, China) containing 80 pancreatic ductal carcinoma and 80 paired non-neoplastic pancreatic tissue samples. TMA was deparaffined and endogenous peroxidase activity was blocked with 3% hydrogen peroxidase. To unmask the immunoepitopes, the slides were immersed in EDTA (1 mmol/L, pH 8.0) and boiled for 15 minutes. The slides were incubated with NDUFAF1 antibody (Abcam, 1:100 dilution) overnightat at 4°C and with secondary antibody at room temperature for 30 mins. Finally, the slides were stained with 3, 3′-diaminobenzidine tetrahydrochloride (DAB) for signal detection and counterstained with 20% hematoxylin. The immunostaining scores were estimated according to the percentage of the staining intensity of the positive cells as described previously [32].

### Flow cytometry analysis of reactive oxygen species (ROS)

Intracellular ROS (H_2_O_2_) contents were measured by incubating cells with 5 μM CM-DCFDA (Invitrogen) at 37°C for 1 h followed by detection using flow cytometry (Beckman Coulter). Intracellular superoxide level was measured by incubating cells with 5 μM MitoSOX (Invitrogen) at 37°C for 1 h before detection by flow cytometry analysis.

### Analysis of oxygen consumption

As previously described [33], 1 million cells were re-suspended in 1 ml culture medium pre-equilibrated with 21% oxygen and placed in a sealed respiration chamber (Oxytherm, Hansatech Instrument) to monitor oxygen consumption rate.

### Metabolic measurements

Glucose uptake and lactate production were measured with Glucose Assay Kit II and Lactate Assay Kit II (Biovision) according to manufacturer's instructions. Aliquots of the medium were removed from cell culture and Glucose uptake and lactate production was determined by the concentration difference between samples and blank medium control. Cellular ATP and NADH contents were measured using the ATP Colorimetric Assay Kit and NADH Quantitation Kit (Biovision) respectively as described before [9].

### Enzyme activity of mitochondria complex I

The activity of mitochondrial complex I was measured using Complex I Enzyme Activity Microplate Assay Kit (Abcam) according to manufacturer's instructions. Briefly, the mitochondrial proteins were extracted and added to the microplate. The complex I enzyme was immune captured within the wells of the microplate, and the activity was determined by the oxidation of NADH to NAD^+^. Complex I activity was measured by the increase in absorbance of the dye at 450 nm and shown as the changes in absorbance per minute.

### Statistical analysis

Statistical difference between NDUFAF1 expression of benign and malignant pancreatic tissue on tissue microarray was analyzed by Fisher's exact test. All other statistical significant difference analyses were performed using a two-tailed Student's *t* test. The data are shown as means ± SD. *P* < 0.05 was considered statistically significant.

## SUPPLEMENTAL METHODS


