# Supplementary material for: Identification of NDUFAF1 in mediating K-Ras induced mitochondrial dysfunction by a proteomic screening approach
Source: Oncotarget. 2015 Feb 27;6(6):3947–62. doi: 10.18632/oncotarget.2968 (PMC4414165; doi:10.18632/oncotarget.2968)
Supplement: Supplementary file 1 [file oncotarget-06-3947-s001.pdf]

SUPPLEMENTAL METHODS

Blue-native Polyacrylamide Gel Electrophoresis (BN-PAGE)

BN-PAGE was performed using a Novex Native PAGE Bis-Tris Gel System (Life Technologies, USA) following manufacturer's instructions. Briefly, mitochondria were isolated, purified and re-suspended in 30 µl of 1 × Native PAGE Sample buffer (Life Technologies, USA) with 1% digitonin and protease inhibitors (Roche, USA). After centrifuge at 20, 000 g for 30 min, 30 µl of the supernatant was re-suspended with 1 µl of 5% G250 sample additive (Life Technologies, USA). 40 µg of mitochondria proteins were loaded on 3–12% Bis-Tris Native PAGE gels. Native unstained protein standard (Life Technologies, USA) was used as molecular weight marker. Protein concentrations of mitochondria was determined by BCA assay (Thermo Scientific, USA).

Measurement of oxygen consumption rate (OCR) by Seahorse XF24

Briefly, cells were plated into XF24 cell culture plates (Seahorse Bioscience, North Billerica) and cultured overnight prior to OCR analysis. T-Rex293 cells before and after K-Ras activation for 24 hrs were seeded at 40,000/well in complete media. The next day, the complete media was replaced with 600 µl Seahorse assay medium(25 mmol/L glucose, 1 mmol/L sodium pyruvate) and cells were cultured at 37°C without CO2 for 1 h. The key parameters of mitochondrial function were directly measured by The XF Cell Mito Stress Test (Seahorse). The compounds (oligomycin, FCCP, and a mix of rotenone and antimycin A) are serially injected to measure ATP production, maximal respiration, and non-mitochondrial respiration, respectively.

SUPPLEMENTARY FIGURES

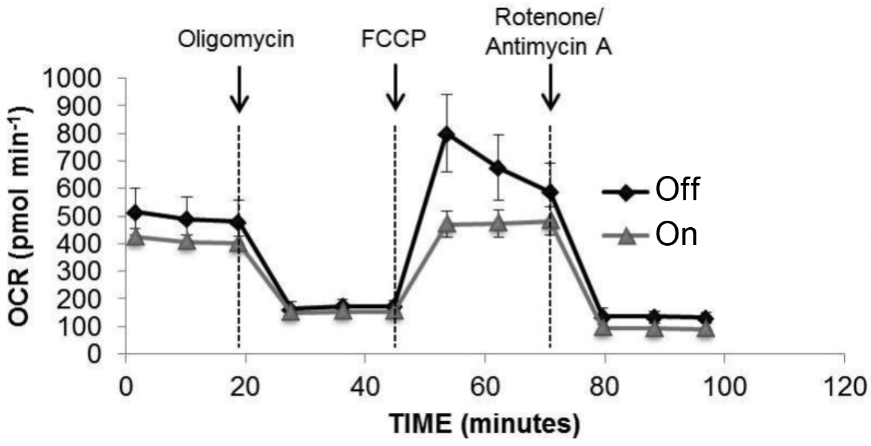

Supplementary Figure 1: Kinetic OCR (oxygen consumption rate) of T-Rex-293 before (Off) and after induction of K-Ras for 48 hrs (On) was analyzed by Seahorse XF analyzer. Oligomycin (1 μM), FCCP (0.5 μM) and Rotenone (1 μM)/ Antimycin A (1 μM) mix were serially injected to measure ATP production, maximal respiration, and non-mitochondrial respiration, respectively. T-Rex-293 off and on (induction for 48 h) cells were plated at 40,000 cells/well 24 hours prior to the assay. Each data point represents mean ± SD, *n* = 3.

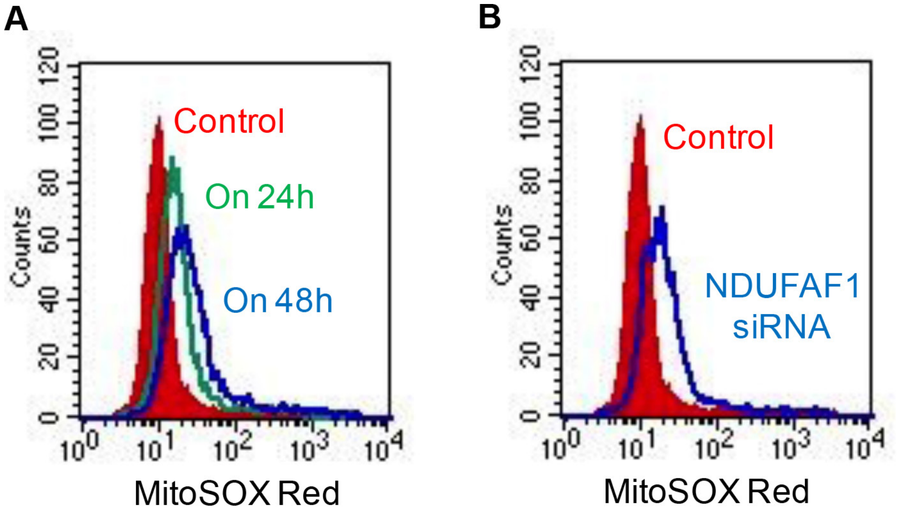

Supplementary Figure 2: Intracellular superoxide generation detected by MitoSOX Red. **(A)** Increase of superoxide generation after K-Ras activation for 24 and 48 hrs. **(B)** Increase of superoxide generation after knockdown of NDUFAF1 by siRNA.

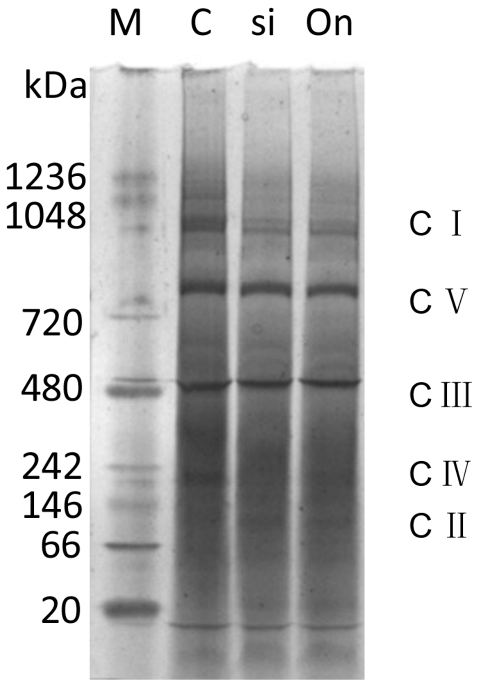

Supplementary Figure 3: BN-PAGE showing assembly of mitochondrial respiratory chain complex of T-ReX293 cells before and after K-Ras activaiton and knockdown of NDUFAF1 by siRNA. M, molecular marker. C, control cells. Si, NDUFAF1 by siRNA. On, induction of K-Ras by doxycyline for 48 hrs. CI-V, complex I to complex V.
